# Traumatic High Cervical Spine Injury Presenting as Cardiac Arrest: A Case Report

**DOI:** 10.7759/cureus.116036

**Published:** 2026-09-10

**Authors:** Iara Ferreira, Joana Reis Aguiar, João Cunha

**Affiliations:** 1 Internal Medicine, Unidade Local De Saúde De Entre Douro E Vouga, Santa Maria da Feira, PRT; 2 Internal Medicine, Unidade Local De Saúde da Arrábida, Setúbal, PRT; 3 Critical Care Medicine, Unidade Local De Saúde De Entre Douro E Vouga, Santa Maria da Feira, PRT

**Keywords:** cardiac arrest, cervical vertebrae injuries, emergency medical services, spinal cord injuries, wounds and injuries

## Abstract

Traumatic high cervical spinal cord injury is a potentially devastating condition that may cause profound cardiovascular and respiratory instability through disruption of descending sympathetic pathways. In severe cases, autonomic dysfunction may contribute to marked hypotension, bradycardia, and cardiac arrest. We report the case of a 47-year-old man who sustained blunt trauma of incompletely characterized mechanism in a workplace accident and was found in cardiac arrest by a prehospital emergency medical team. Advanced life support was initiated, and return of spontaneous circulation was achieved after approximately eight minutes. The documented arrest rhythms included pulseless electrical activity and pulseless ventricular tachycardia. On admission, the patient remained intubated and mechanically ventilated and developed marked hypotension and bradycardia requiring norepinephrine support. Extended focused assessment with sonography for trauma showed no evidence of pneumothorax, pericardial effusion, or free intraperitoneal fluid. Computed tomography revealed a severe C2-C3 distraction injury with intersomatic avulsion, disruption of the posterior ligamentous complex, extensive traumatic hematoma involving the vertebral and spinal canal regions, and associated spinal cord injury. Following multidisciplinary assessment, the injuries were considered irreversible, with no feasible surgical or other outcome-modifying therapeutic option, and care was transitioned to comfort measures. The patient died approximately four and a half hours after admission. This case highlights the potential for severe high cervical spinal cord injury to present with profound autonomic cardiovascular instability and cardiac arrest and emphasizes the importance of considering occult cervical spinal trauma in cases of unexplained cardiorespiratory collapse following major trauma.

## Introduction

Traumatic spinal cord injury (SCI) is a devastating neurological condition associated with substantial mortality, long-term disability, and systemic complications. The initial mechanical insult causes direct disruption of neural and vascular structures and is followed by a secondary injury cascade involving ischemia, edema, hemorrhage, excitotoxicity, oxidative stress, and neuroinflammation, which may progressively extend spinal cord damage beyond the primary lesion [1,2]. Cervical injuries are particularly critical because they can simultaneously compromise motor and sensory pathways, respiratory function, and autonomic cardiovascular control.

Cardiovascular instability is a well-recognized complication of acute cervical SCI. Interruption of descending supraspinal sympathetic pathways reduces sympathetic outflow while cardiac parasympathetic activity remains relatively preserved. This autonomic imbalance results in peripheral vasodilation, reduced venous return, hypotension, and bradycardia, with the severity of cardiovascular dysfunction generally increasing with higher and more extensive spinal cord lesions [3]. Severe bradyarrhythmias, asystole, and cardiac arrest have been described following traumatic cervical SCI and may represent life-threatening manifestations of profound autonomic dysfunction [3,4].

Respiratory impairment may further contribute to clinical deterioration. High cervical SCI can compromise diaphragmatic and accessory respiratory muscle function, impair ventilation and cough effectiveness, and predispose patients to acute respiratory failure and hypoxemia [5]. Consequently, the acute management of cervical SCI requires rapid recognition of the injury, appropriate spinal stabilization, adequate oxygenation and ventilation, and meticulous hemodynamic management. Prevention of systemic hypotension is particularly important because reduced spinal cord perfusion may exacerbate secondary ischemic injury. Contemporary clinical practice guidelines therefore emphasize early hemodynamic monitoring and blood pressure augmentation in patients with acute traumatic SCI [6].

Although cardiovascular complications following cervical SCI are well established, cardiac arrest as an early or presenting manifestation is uncommon and may obscure the underlying spinal injury, particularly when the traumatic mechanism is unclear or the neurological examination is limited [4,7]. Recognition of this association is therefore clinically important when evaluating otherwise unexplained cardiorespiratory collapse following trauma. The autonomic mechanisms underlying cardiovascular dysfunction after SCI have been extensively described, while current clinical practice guidelines recommend that early decompressive surgery within 24 hours be offered as an option for adults with acute SCI when clinically appropriate [8,9]. We report a case of severe traumatic C2-C3 distraction injury presenting with cardiac arrest followed by profound hypotension and bradycardia after return of spontaneous circulation (ROSC), highlighting the potentially catastrophic autonomic consequences of high cervical SCI.

## Case presentation

A 47-year-old man was assessed by a prehospital emergency medical team following a workplace accident involving incompletely characterized blunt trauma. According to the available information, a heavy load may have fallen onto or compressed the cervical, cervicothoracic, facial, and abdominal regions. The exact mechanism of injury could not be established because the patient had already been moved before the arrival of the emergency team.

On initial assessment, the patient was found in cardiac arrest of unknown duration. Advanced life support was immediately initiated, including endotracheal intubation. The documented arrest rhythms included two episodes of pulseless electrical activity and two episodes of pulseless ventricular tachycardia. ROSC was achieved after approximately eight minutes of advanced life support. During prehospital resuscitation and stabilization, the patient received 3 L of intravenous crystalloid solution and 1 g of tranexamic acid before transport to the emergency department. No sedative or analgesic medication was administered following ROSC.

On arrival at the emergency department, the patient remained intubated and mechanically ventilated. Bilateral breath sounds were present, with no clinically apparent thoracic instability. He was markedly hypotensive and bradycardic, requiring hemodynamic support with a norepinephrine infusion. Extended focused assessment with sonography for trauma (eFAST) showed no evidence of pneumothorax, pericardial effusion, or free intraperitoneal fluid. The major clinical events, diagnostic findings, and therapeutic interventions are summarized in Table 1.

**Table 1:** Timeline of the clinical course, diagnostic findings, and management ALS, advanced life support; CT, computed tomography; eFAST, extended focused assessment with sonography for trauma; ROSC, return of spontaneous circulation

Clinical phase	Main findings/events	Management
Workplace accident	Incompletely characterized blunt trauma, possibly involving compression of the cervical, cervicothoracic, facial, and abdominal regions	Prehospital emergency medical team activated
Initial prehospital assessment	Cardiac arrest of unknown duration; documented rhythms included two episodes of pulseless electrical activity and two episodes of pulseless ventricular tachycardia	Advanced life support; endotracheal intubation
After approximately 8 min of ALS	ROSC achieved	Continued stabilization; 3 L intravenous crystalloid solution and 1 g tranexamic acid administered
Transport to emergency department	No sedative or analgesic medication administered after ROSC	Transfer under advanced medical support
Emergency department admission	Intubated and mechanically ventilated; bilateral breath sounds present; no clinically apparent thoracic instability; marked hypotension and bradycardia	Norepinephrine infusion initiated
Initial trauma assessment	eFAST showed no evidence of pneumothorax, pericardial effusion, or free intraperitoneal fluid	Continued hemodynamic and ventilatory support
Cervical CT	Severe C2-C3 traumatic distraction injury with intersomatic avulsion, disruption of the posterior ligamentous complex, extensive vertebral/spinal canal hematoma, and findings consistent with spinal cord injury	Multidisciplinary assessment
Multidisciplinary discussion	The extent of the traumatic injury was considered irreversible; no surgical or other potentially outcome-modifying therapeutic option was considered feasible	Decision to limit further invasive or life-prolonging treatment and transition to comfort-focused care
Final outcome	Family informed of the severity of the injuries and unfavorable prognosis and allowed to remain with the patient	Comfort measures; death approximately 4.5 h after hospital admission

Computed tomography (CT) of the cervical spine revealed a severe high cervical traumatic injury. Coronal and sagittal CT reconstructions demonstrated marked distraction at the C2-C3 level, with intersomatic avulsion and severe disruption of normal cervical alignment (Figure 1).

**Figure 1:**
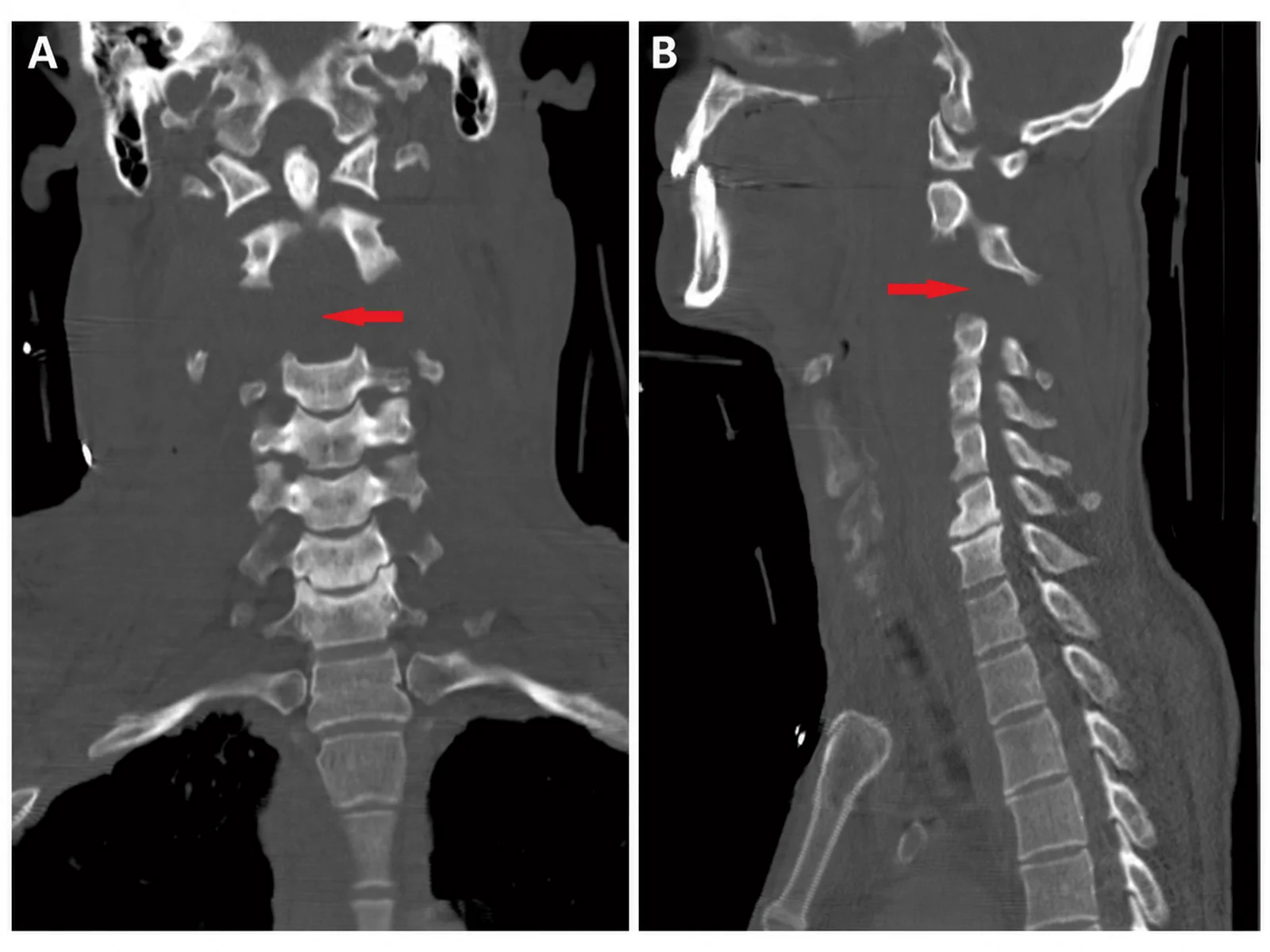
Coronal and sagittal CT reconstructions of the cervical spine demonstrating severe C2-C3 distraction injury (A) Coronal reconstruction demonstrating marked separation at the C2-C3 level (red arrow). (B) Sagittal reconstruction demonstrating C2-C3 intersomatic avulsion and disruption of normal cervical alignment (red arrow). CT, computed tomography

Three-dimensional CT reconstruction further illustrated the marked separation and displacement between C2 and C3 associated with the traumatic injury (Figure 2).

**Figure 2:**
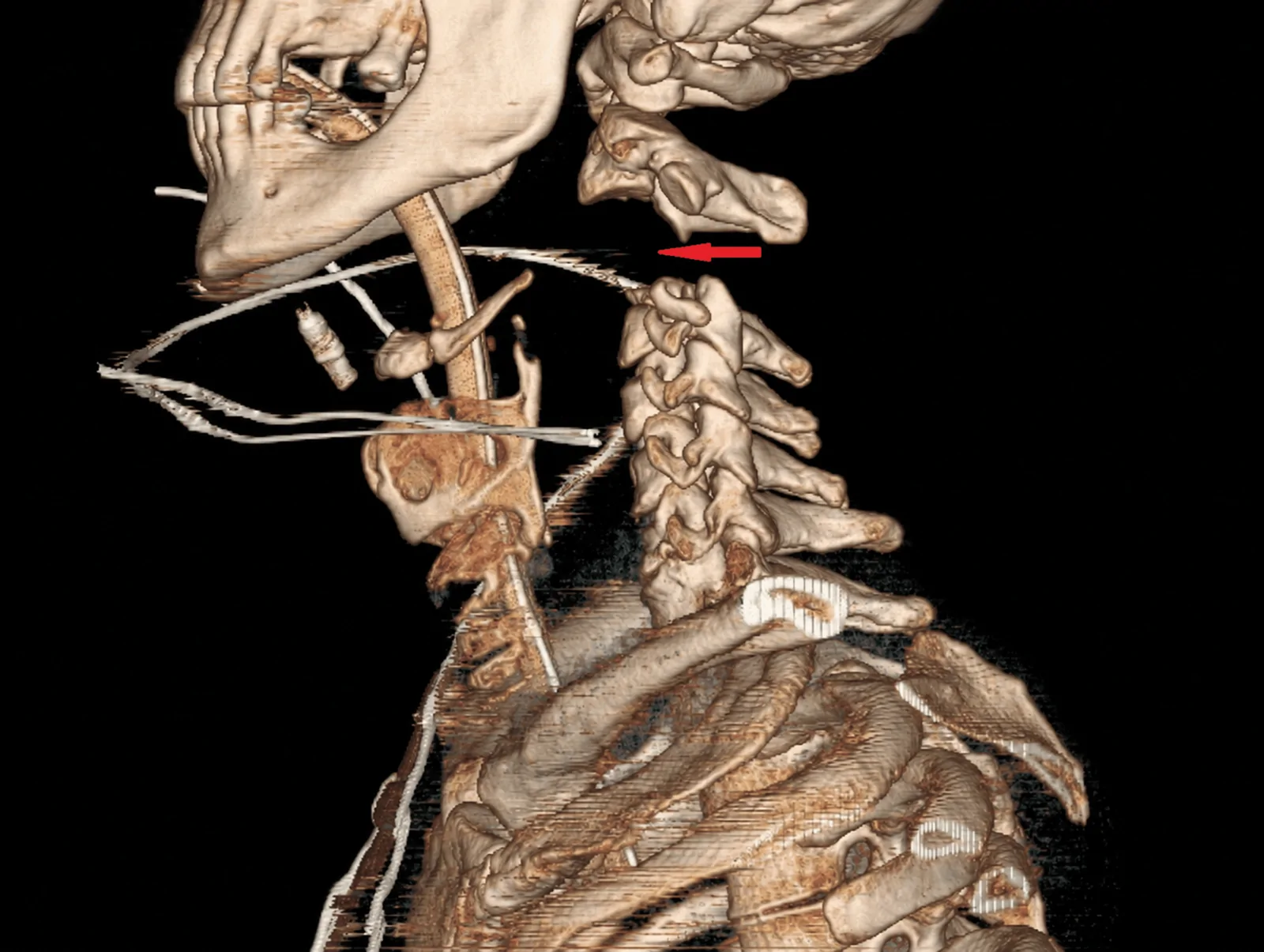
Three-dimensional CT reconstruction of the severe C2-C3 traumatic injury Three-dimensional reconstruction demonstrating marked separation and displacement between C2 and C3 (red arrow). CT, computed tomography Image created using Volume Viewer (GE HealthCare, Chicago, IL, USA).

Additional imaging findings included disruption of the posterior ligamentous complex, extensive traumatic hematoma involving the vertebral and spinal canal regions, and findings consistent with associated spinal cord injury.

Following review of the imaging findings, the case was discussed by a multidisciplinary team comprising intensive care physicians, orthopedic surgeons, neurologists, and surgeons. The extent of the traumatic injury was considered irreversible, and no surgical or other potentially outcome-modifying therapeutic option was considered feasible. Further invasive or life-prolonging interventions were therefore considered nonbeneficial, and the decision was made to transition to comfort-focused care.

The patient's family was fully informed of the severity and irreversible nature of the injuries, the extremely poor prognosis, and the expected unfavorable outcome. They were allowed to remain with the patient throughout end-of-life care. The patient subsequently died approximately four and a half hours after hospital admission.

## Discussion

This case illustrates a severe traumatic high cervical spinal cord injury presenting with cardiac arrest, followed by persistent bradycardia and profound hypotension after ROSC. Although a direct causal relationship between the spinal injury and the initial cardiac arrest cannot be definitively established, the anatomical level and severity of the C2-C3 injury, together with the subsequent cardiovascular pattern, strongly support a clinically relevant contribution from acute autonomic dysfunction. Cardiovascular disturbances are particularly common after cervical spinal cord injury because disruption of descending sympathetic pathways can result in loss of sympathetic vascular tone and relative parasympathetic predominance [3,4,8].

The cardiovascular consequences of acute spinal cord injury depend substantially on the neurological level and completeness of the lesion. Supraspinal sympathetic pathways descend through the cervical spinal cord before reaching sympathetic preganglionic neurons, and interruption of these pathways in high cervical lesions may markedly reduce sympathetic outflow. The resulting peripheral vasodilation, reduction in venous return, and impaired chronotropic response can produce the characteristic combination of hypotension and bradycardia associated with neurogenic shock [8]. Bradyarrhythmias are particularly frequent during the acute phase of cervical spinal cord injury, and severe cases may progress to profound bradycardia or cardiac arrest [3,4]. In the present patient, persistent hypotension and bradycardia after resuscitation, requiring norepinephrine support, were therefore compatible with significant autonomic cardiovascular impairment secondary to the high cervical injury.

Cardiac arrest as an initial or early manifestation of cervical spinal cord injury is uncommon and presents an important diagnostic challenge. Published evidence remains largely limited to individual case reports and small case series. Across the five closely related reports considered here, eight patients with cardiac arrest occurring in association with cervical spinal injury have been described [4,7,10-12]. Kim et al. reported a patient with traumatic cervical spinal cord injury involving C4-C7 who developed cardiac arrest during hospitalization in the setting of severe autonomic dysfunction. The patient was successfully resuscitated and subsequently underwent permanent pacemaker implantation, with no further cardiac events [4]. Nissen et al. described two patients with cervical fractures and associated spinal cord injury who presented with cardiac arrest after apparently low-impact cycling accidents. One patient died after life-sustaining treatment was withheld because of severe neurological injury, whereas the second underwent surgical stabilization and ultimately achieved full recovery [10]. Mayà-Casalprim et al. reported two patients in whom traumatic cervical spinal cord injury was initially overlooked after out-of-hospital asystolic cardiac arrest; both patients subsequently died [7]. Hawayek et al. described a patient who was found pulseless after a motor vehicle accident and was subsequently diagnosed with C2-C3 fracture-dislocation and spinal cord compression. She underwent decompression and C1-C5 fusion and survived with substantial neurological recovery [11]. More recently, Chung et al. described two patients with out-of-hospital cardiac arrest in whom cervical spine injury was initially unsuspected because the traumatic mechanism was unclear; both died during hospitalization [12]. Within this small and heterogeneous group of eight published patients, five died and three survived. However, differences in injury mechanism, neurological level and severity, associated pathology, duration of cardiac arrest, timing of cardiac arrest relative to the injury, and available therapeutic options preclude meaningful quantitative comparison of outcomes [4,7,10-12].

These reports emphasize that cervical spinal trauma may be overlooked when cardiac arrest dominates the initial clinical presentation, particularly when the traumatic mechanism is uncertain or neurological assessment is limited [7,10-12]. In our patient, the exact mechanism of injury was incompletely characterized because he had been moved before the arrival of the prehospital team. Moreover, cardiac arrest preceded definitive identification of the cervical lesion, making early etiological assessment particularly difficult.

The arrest rhythms documented in this case included both pulseless electrical activity and pulseless ventricular tachycardia. This combination precludes attribution of the arrest to a single pathophysiological mechanism. Nevertheless, the subsequent development of marked bradycardia and hypotension in the presence of a catastrophic C2-C3 injury provides important physiological support for an autonomic component. The negative eFAST examination, with no evidence of pneumothorax, pericardial effusion, or free intraperitoneal fluid, also meant that these major immediately identifiable traumatic causes of circulatory collapse were not demonstrated during the initial assessment. However, a negative eFAST does not exclude all potential traumatic or post-resuscitation causes of hemodynamic instability. The association between the cervical injury and cardiac arrest should therefore be interpreted within the overall clinical context rather than as evidence of exclusive causality.

Respiratory dysfunction represents an additional mechanism by which high cervical spinal cord injury may contribute to cardiorespiratory deterioration. Cervical spinal cord lesions can impair diaphragmatic function, accessory respiratory muscle recruitment, cough effectiveness, and ventilation, with higher lesions carrying the greatest risk of respiratory failure [5]. In catastrophic high cervical trauma, simultaneous impairment of respiratory and autonomic cardiovascular control may therefore result in rapid clinical deterioration. In this case, endotracheal intubation and mechanical ventilation were initiated during advanced life support, limiting the ability to determine the independent contribution of respiratory failure to the initial cardiac arrest. Nevertheless, the anatomical severity of the lesion makes respiratory compromise physiologically plausible.

The imaging findings were particularly striking. Coronal and sagittal CT reconstructions demonstrated marked C2-C3 distraction with intersomatic avulsion and severe disruption of normal cervical alignment, while three-dimensional reconstruction clearly illustrated the extensive separation and displacement between the vertebral segments. Associated disruption of the posterior ligamentous complex, extensive traumatic hematoma involving the vertebral and spinal canal regions, and spinal cord injury further demonstrated the destructive nature of the trauma. These findings were consistent with a highly unstable high cervical injury and provided an anatomical explanation for the profound neurological and autonomic consequences observed.

Hemodynamic stabilization is a major component of the early management of acute traumatic SCI because systemic hypotension may further reduce spinal cord perfusion and aggravate secondary ischemic injury [1,2]. The most recent clinical practice guideline suggests actively augmenting mean arterial pressure to a lower limit of approximately 75-80 mmHg while avoiding active augmentation beyond 90-95 mmHg, generally for three to seven days, although these recommendations are based on very low-quality evidence [6]. In the present case, norepinephrine was required because of profound post-resuscitation hypotension. However, the severity of the structural injury and the resulting prognosis ultimately precluded application of a longer-term spinal cord perfusion strategy.

Early surgical decompression is generally recommended as an option for adults with acute spinal cord injury when neurological salvage is considered possible, with contemporary guidelines supporting decompression within 24 hours of injury [9]. This principle must nevertheless be individualized according to injury severity, neurological status, associated injuries, physiological stability, and the feasibility of meaningful neurological recovery. In the present case, the imaging findings and overall clinical condition were reviewed by a multidisciplinary team involving intensive care, orthopedic, neurological, and surgical specialists. The injury was considered irreversible, and no surgical or other potentially outcome-modifying intervention was considered feasible. The transition to comfort-focused treatment therefore followed multidisciplinary assessment of the severity of the injury and its prognosis rather than failure to pursue an otherwise indicated surgical strategy.

This case has several limitations. The exact traumatic mechanism and duration of cardiac arrest before arrival of the emergency medical team were unknown, and the presence of multiple arrest rhythms prevents definitive identification of a single mechanism for the cardiac arrest. Furthermore, the extreme clinical severity and rapid evolution of the case limited detailed neurological characterization and additional diagnostic investigations. Somatosensory evoked potential studies were not performed. In previously reported patients with initially unrecognized cervical spinal cord injury after cardiac arrest, median nerve somatosensory evoked potentials demonstrated bilateral absence of the cortical N19 responses together with abnormalities of the cervicomedullary N13 responses, providing complementary neurophysiological evidence of cervical pathway dysfunction [7]. Such studies may contribute to neurological characterization and prognostic assessment when the clinical examination is limited, but their findings must be interpreted together with the neurological examination, imaging, and overall clinical context rather than being considered an isolated marker of irreversible spinal cord injury. In the present case, the absence of somatosensory evoked potential testing therefore limits neurophysiological characterization of the lesion but does not alter the extensive structural abnormalities demonstrated on imaging and considered during multidisciplinary assessment. Consequently, it cannot be stated with certainty that autonomic dysfunction caused the initial cardiac arrest. Nevertheless, the combination of catastrophic C2-C3 spinal injury, absence of major abnormalities on eFAST, and profound post-resuscitation bradycardia and hypotension provides a coherent clinical and physiological association.

The principal learning point from this case is that severe high cervical spinal cord injury should be considered in patients presenting with unexplained cardiorespiratory collapse following trauma, particularly when the mechanism of injury is uncertain. Recognition of the association between cervical spinal trauma, autonomic dysfunction, respiratory compromise, and cardiac arrest may facilitate earlier diagnosis, appropriate imaging, and timely multidisciplinary decision-making.

## Conclusions

Severe high cervical spinal cord injury may cause profound autonomic and respiratory dysfunction and, in extreme cases, may contribute to cardiac arrest. This case highlights the importance of considering occult cervical spinal trauma in patients presenting with unexplained cardiorespiratory collapse after major trauma, particularly when the mechanism of injury is unclear and the initial neurological assessment is limited. Persistent bradycardia and hypotension after return of spontaneous circulation should raise suspicion for significant autonomic impairment in the appropriate clinical context. Early cervical imaging, aggressive initial stabilization, and prompt multidisciplinary assessment are essential to establish the diagnosis, evaluate therapeutic options, and guide prognosis. Although a direct causal relationship between the C2-C3 injury and the initial cardiac arrest cannot be definitively proven, the clinical and imaging findings strongly support a major contribution from the catastrophic high cervical injury.
